# Extraneuronal Monoamine Transporter Mediates the Permissive Action of Cortisol in the Guinea Pig Trachea: Possible Involvement of Tracheal Chondrocytes

**DOI:** 10.1371/journal.pone.0076193

**Published:** 2013-10-01

**Authors:** Chen Wang, Wenying Qiu, Yiqing Zheng, Hui Li, Yijia Li, Bing Feng, Shu Guo, Li Yan, Ji-Min Cao

**Affiliations:** 1 Department of Medicine, Peking Union Medical College, Chinese Academy of Medical Sciences, Beijing, China; 2 Department of Anatomy, Histology and Embryology, Institute of Basic Medical Sciences Chinese Academy of Medical Sciences, School of Basic Medicine Peking Union Medical College, Beijing, China; 3 Department of Physiology and Pathophysiology, Institute of Basic Medical Sciences Chinese Academy of Medical Sciences, School of Basic Medicine Peking Union Medical College, Beijing, China; Research Center Borstel, Germany

## Abstract

Cortisol, a member of glucocorticoids, could potentiate the action of catecholamine by a non-genomic mechanism. Although this permissive effect has been well appreciated in the anti-asthmatic medication, the underlying signaling pathway has remained mysterious. Here, we show that extraneuronal monoamine transporter (EMT), a membraneous reuptake transporter for circulating catecholamine clearance, is the direct target of cortisol in its permissive effect. We found that BSA-conjugated cortisol, which functions as a cortisol but cannot penetrate cell membrane, enhanced the spasmolytic effect of β-adrenoceptor agonist (isoprenaline) in histamine-sensitized tracheal spirals of guinea pigs, and pharmacological inhibition of EMT with famotidine was powerful enough to imitate the permissive action of cortisol. To our surprise, EMT protein expression was high in the chondrocytes of tracheal cartilage, but was undetectable in tracheal smooth muscle cells. The functionality of EMT was further confirmed with measurement of catecholamine uptake by tracheal chondrocytes. Moreover, cortisol-initiated membrane signaling could activate protein kinase C (PKC), which phosphorylates EMT and induces its internalization via a lipid raft-dependent pathway. Both of the mechanisms slow down the reuptake process by chondrocytes, leading to extracellular catecholamine accumulation and results in a more profound adrenergic signaling activation in tracheal smooth muscle cells. Thus, an EMT-centered pathway was proposed to explain the permissive action of cortisol. Collectively, our results highlight the role of EMT in the crosstalk between glucocorticoid and catecholamine. EMT may represent a promising target for adrenergic signaling modulation.

## Introduction

As neurotransmitters and hormones, catecholamines occupy a key position in the regulation of various physiological and pathological processes. Once released from stored vesicles, catecholamines interact with adrenergic receptors to produce characteristic responses of the effecter. Unlike acetylcholine, which is degraded by powerful cholinesterase at the site of cholinergic transmission, the action of catecholamines is terminated by reuptake and metabolic transformation. It has been known for many years that neurons adopt a sodium-dependent process for catecholamines removal. In contrast, clearance of circulating catecholamines is primarily by nonneuronal mechanisms with distinct pharmacological profiles [1]. The presence of extraneuronal monoamine transporter (EMT) was first noticed by a fortuitous observation in 1965 [2]. Relative to classical norepinephrine transporter (NET), EMT was defined as an extraneuronal transporter with low affinity, high maximum rate and epinephrine preference. The physiological relevance of this transporter was highlighted when its genetic locus was identified as a strong susceptible site for coronary artery disease in a genome-wide haplotype (GWHA) study [3]. However, its participation and regulation in other adrenergic processes, especially airway disorders, is still obscure.

In early biochemical characterization, EMT was shown to be inhibited by a series of steroid hormones in a dose-dependent manner [4]. This unique property distinguishes EMT from NET, in which NET is sensitive to desipramine and cocaine. This unexpected regulation seems to indicate a potential crosstalk between steroids and catecholamine signaling. Indeed, steroids have been demonstrated to enhance the responses of vascular smooth muscles to catecholamines, which were attributed to catechol-O-methyltransferase (COMT) inhibition in an early assumption [5]. In respect of EMT inhibition, steroids ought to potentiate catecholamine signaling in diverse physiological processes.

Cortisol, a typical member of glucocorticoids, has attracted tremendous attentions in biomedical research, due to its diverse functions. Classical effects of cortisol are mediated by cytosolic glucocorticoid receptor (GR) via translocation into the nucleus and regulation of gene expression. Cortisol can also exert rapid, transcription-independent actions in seconds or minutes after its application, as the permissive action. These so called “non-genomic effects” have been noted at least half-century and are true for near all steroids. Non-genomic effect does not influence gene expression, but drives more rapid biological actions via specific receptors localized most often to the plasma membrane. Although traditional G protein-coupled receptors (GPCRs) are thought to be the effecter for these actions, other membrane proteins with well-defined functions can also be intelligible candidates if they bear steroid-binding sites. In fact, this is the case for progesterone, which stimulates acrosomal reaction via activates a sperm specific calcium channel (CatSper) [6,7]. So far, no such a molecule has been well-characterized for the non-genomic effect of glucocorticoids in a specialized physiological or pathological condition.

We hypothesize that EMT is the direct target of glucocorticoid in its permissive action. Glucocorticoids may inhibit the function of EMT by certain means and therefore slows down the process of catecholamine reuptake, giving rise to more catecholamines accumulation around the effecter and results in augmentation of downstream signaling. Indeed, the observation that glucocorticoid potentiates the effect of catecholamine on trachea relaxation has been made across several species [8,9]. And this potentiation seems to be related to decreased catecholamine uptake by smooth muscle rich parts of the trachea [10,11]. This interaction can be particularly meaningful, since both glucocorticoids and catecholamines are blood-borne bioactive molecules involved in numerous physiological events and the importance of this interaction can also be exemplified from a clinical point of view. Among anti-asthmatic drugs, the combination of inhaled glucocorticoids and β-agonists is the most potent and effective medication for asthma control. This appreciated action is not only due to the complementary effects of these two agents on the pathogenesis of asthma, but also attributed to enhanced bronchodilatation via the permissive action as we showed recently [12] and studies from other groups [13,14].

To address the hypothesis mechanistically, we utilized guinea pig tracheal spiral, a well-recognized *ex vivo* model of permissive action, in which glucocorticoids have been shown to potentiate catecholamine-induced relaxation [8]. After confirmed the action of cortisol in this model was mediated by “receptors” localized to the plasma membrane, we found that EMT was highly enriched in chondrocytes, rather than the smooth muscle cells, of guinea pig trachea. The function of this transporter was further justified with the use of EMT inhibitors and catecholamine uptake was measured by a semiquantitative fluorescent method in the chondrocytes of tracheal cartilage. Thus, the modulation on EMT seemed to be powerful enough to realize the permissive action, which was independent of the classical signaling of glucocorticoids on transcription regulation. Further pharmacological and bioinformatics analyses revealed possible post-translational modification and downregulation of membranous EMT, which might also account for the permissive action to a certain extent. With the coordination of published genetic data, we noticed a potential association between glucocorticoid nongenomic pathway and airway disorders. Our observations indicate that cartilaginous EMT plays a critical role in tracheal catecholamine metabolism and function. This finding may trigger more studies to explore the metabolic function of cartilage. These results also provide a fundamental insight for the crosstalk between glucocorticoids and catecholamines and have conceptual implications concerning the utility of EMT modulators for adrenergic signaling regulation.

## Materials and Methods

### Animals

Young Hartley guinea pigs (250–350 g, 2–3 month-old) with no restriction to gender were used. The animals were housed under room temperature (22–26°C) and maintained in a 12:12 light-dark cycle. Regular food and water were free access. The animal use protocol was approved by the Life Ethics Committee of Peking Union Medical College and was conducted in compliance with the U.S. National Institutes of Health Guidelines for the Care and Use of Laboratory Animals (NIH Publication 85-23).

### Reagents

Reagents were purchased from the following suppliers: cortisol (Tianjin Jinyao Amino Acid Co. Ltd., Tianjin, China), histamine (Sinopharm Chemical Reagent Co. Ltd., Shanghai, China), isoprenaline (Shanghai Harvest Pharmaceuticals Co. Ltd., Shanghai, China), BSA-cortisol (Sigma-Aldrich, stoichiometry 1: 25), famotidine (Shanghai Sine Pharmaceutical Co. Ltd., Shanghai, China), phorbol-12-myristate-13-acetate (PMA) (Beyotime, Jiangsu, China), okadaic acid (Beyotime, Jiangsu, China), filipin III (Sigma-Aldrich), concanavalin A (ConA) (Sigma-Aldrich).

### Cell culture

Rat vascular smooth muscle (VSM) cells were prepared from the aorta of adult SD rats as described previously [15] with minor modifications. Briefly, the thoracic aorta was removed along with the aortic arch. Vessels were stripped of adventitia and cut longitudinally, and the endothelium was removed by scraping with scissors. Tissue was cut into pieces (1 mm^2^) and placed intima side down in culture flasks. Dulbecco’s Modified Eagle Medium (DMEM) with 10% fetal bovine serum (FBS) was added, and the flasks were placed in a humidified incubator at 37°C with 5% CO_2_-95% air. After 4–7 days, the cells began to migrate out from the tissue sections, reaching confluence in 2–3 weeks. Upon confluence, the cells were detached with 0.05% trypsin-0.02% EDTA and seeded on culture plates (passage 1). Cells were then split 1:3 approximately every 3 days. Cells at passage 3-8 were used for further experiments.

### Immunohistochemical and immunofluorescent staining

Trachea harvested from guinea pig were fixed in 10% formalin, dehydrated in graded alcohol, cleared in xylene, and embedded in paraffin. Sections with 5 mm in thickness were cut and mounted onto slide and routinely developed. Immunohistochemical staining of EMT was performed with a standard ABC method [16]. Briefly, tissue sections were incubated with antibody against EMT (P-16, sc-18515) (1:50, Santa Cruz Biotechnology, Santa Cruz, CA, USA), followed by incubations with biotinylated secondary antibodies (Santa Cruz) and ABComplex/HRP (Santa Cruz) at room temperature. The sections were washed with PBS between each staining. The immunoreactive products were visualized by liquid DAB chromogen (Santa Cruz). The sections were then developed and mounted.

For immunofluorescent labeling, trachea was fixed in the same fixative and then cryoprotected in 30% sucrose overnight. The trachea was frozen and sectioned at 6–8 μm thick on a cryostat. Sections were incubated with blocking buffer (10% normal horse serum and 0.2% Triton X-100 in PBS) for 1h, followed by overnight incubation with primary antibody (goat anti-EMT, 1:50, Santa Cruz) at room temperature and then with secondary antibody (Alexa Fluor 488-conjugated donkey-anti-goat, 1:400, Invitrogen) for 1h. DAPI (4',6-Diamidino-2-Phenylindole) was used to stain nuclear profiles. The labeling specificity for primary antibody was further validated by preabsorption with corresponding antigen (20 μg/ml) (sc-18515 P, Santa Cruz) for 2 h at room temperature prior to the use in immunofluorescence.

Vascular smooth muscle cells grown on glass coverslips were treated with different agents. PMA (10 μM) was used to activate PKC. Filipin III (5 μg/ml) was added 20 min before PMA treatment to evaluate the role of lipid raft/caveolin-mediated endocytosis. After treatment, cells were washed twice with ice-cold PBS, fixed with 4% paraformaldehyde for 20min at room temperature and processed for immunofluorescent staining with standard method as described above.

### Sucrose-potassium phosphate-glyoxylic acid (SPG) method for catecholamine visualization and quantitation

Guinea pig tracheas were isolated and incubated in the tissue chamber with oxygenated Krebs-Henseleit (K-H) buffer solution, as described in the section *Measurement of tracheal smooth muscle tension* below. After 30-min equilibrium, tracheas were treated with cortisol (1 μM) or famotidine (50 μM) for 5 min. In consideration of the sensitivity of aldehyde-based histochemical method to detect intracellular catecholamine, norepinephrine (50 μM) was used as the substrate for EMT uptake. The saline solution of norepinephrine contained 0.1% ascorbic acid to prevent oxidation. After 20 min incubation, tracheas were washed with fresh K-H buffer solution for 10 min to eliminate the norepinephrine bound with the intercellular matrix [17]. Then tracheas were embedded with optimal temperature cutting compound, rapidly frozen in liquid nitrogen and sectioned (10 μM thick) with a cryostat. Intracellular catecholamine was measured using a sucrose-potassium phosphate-glyoxylic acid (SPG) method described for tissue slices [18]. To prevent the time-dependent decaying of intracellular catecholamine, immediately after the sectioning, tracheal sections were dipped into SPG solution (0.2 M sucrose, 0.236 M KH_2_PO_4_ and 1% glyoxylic acid monohydrate, titrated to pH 7.4 with 1 N NaOH) at room temperature for 3 sec. After 5-min air-drying, the specimen were covered with mineral oil and sealed with coverslip, heated at 95°C for 2.5 min, and immediately followed by microscopic visualization of SPG fluorescence with an Olympus fluorescence microscope (BX61) equipped with filters designed for catecholamine fluorescence visualization. A wavelength of 405 nm was used for excitation and the emission was measured at > 455 nm. All sections were photographed with a digital camera at the same magnification (40× objective lens) [19]. For quantitation of catecholamine level in the tissue, the fluorescence intensity in chondrocytes and tracheal smooth muscle cells was measured under high power field (40× objective lens). The fluorescence-positive cells (mostly chondrocytes in this study) on a slide were measured by selecting different, well-separated regions on the same slide. The mean fluorescent intensity of each cell was measured with the cellSens software (Olympus Inc.) and expressed in arbitrary units and corrected with background fluorescence. Average catecholamine uptake of each group was calculated using mean normalized fluorescent intensity of all cells (approximately 50 cells in each group) [20].

SPG method to evaluate catecholamine uptake in cultured cells was performed as previously described [20] with minor modifications. Briefly, vascular smooth muscle cells grown on glass coverslips were equilibrated in incubation solution (137 mM NaCl, 10 mM NaHCO_3_, 0.2 mM NaH_2_PO_4_, 5.4 mM KCl, 0.5 mM KH_2_PO_4_, 6 mM glucose, 0.15 mM CaCl_2_, 2 mM MgCl_2_, 10 mM *N*-2-hydroxyethylpiperazine-*N*-ethane sulfonic acid, and 0.02% bovine serum albumin, pH 7.0) for 30 min at 37°C. For catecholamine uptake assay, cells were exposed to a defined norepinephrine concentration (50 μM) with or without different inhibitors. The saline solution of norepinephrine contained 0.1% ascorbic acid to prevent oxidation. Cortisol (1 μM) or famotidine (50 μM) were added 5 min before norepinephrine exposure. At the end of incubation period, cells were washed with high KCl (20 mM) containing incubation solution and cooled down at 4°C for 10 min. Coverslips with cells were further processed with SPG method to evaluate catecholamine uptake as described above.

### Measurement of tracheal smooth muscle tension

The animals were sacrificed by cervical dislocation, and the trachea was removed as soon as possible under room light during light phase. Isolated trachea was sunk in prewarmed Krebs-Henseleit (K-H) buffer solution (37°C), and the connective tissues were eliminated. Tracheal spiral was prepared as described elsewhere [21]. The tracheal spiral was put onto a tissue chamber and perfused with K-H buffer solution containing (in mM): NaCl 118, KCl 4.7, MgSO_4_·7H_2_O 3.4, CaCl_2_·2H_2_O 2.52, NaHCO_3_ 24.48, KH_2_PO_4_ 1.18, glucose 11, oxygenated continuously at 37°C. Before experiment, the smooth muscle spiral was preloaded with a 1 g tension initially and then stabilized for approximately 30 min. During the stabilization period, the spiral was washed every 10 min. Histamine (10 μM) was used to contract the tracheal smooth muscles. Mechanical tension was recorded by the BL-420E data acquisition system through a force transducer (Chengdu Technology & Market Co. Ltd., Chengdu, China). Testing agents (cortisol, 150 μM; famotidine 50 μM; BSA-cortisol, 840 ng/ml, 1640 ng/ml and 2520 ng/ml; PMA, 10 μM; okadaic acid, 5 nM) were added before induction of tracheal relaxation by β-agonist isoprenaline (0.1 μM). Filipin III (an inhibitor of caveolin-dependent endocytosis) (5 μg/ml) or concanavalin A (ConA, an inhibitor of clathrin-dependent endocytosis) (0.25mg/ml) was used to estimate the role of EMT internalization in the permissive action. PMA was used to evaluate the involvement of PKC-mediated EMT phosphorylation in the permissive action.

### Therapeutic index calculation

Therapeutic index is defined as “b/a”, which has been reported previously as “percentage inhibition of maximal contraction” [22]. In this formula, “a” indicates maximal contraction induced by histamine, while “b” refers to relaxation induced by β-agonist. The therapeutic value was normalized with the control group in each experimental set to eliminate the differences in responsiveness.

### Molecular modeling

Three-dimensional structure of guinea pig EMT was produced by RaptorX web server (raptorx.uchicago.edu) and the models were drawn using PYMOL [23].

### Microarray dataset

To explore whether EMT may express in a circadian manner, the temporal expression profile of *EMT* was extracted from a published microarray dataset (GEO accession GSE 11922), which was established with the use of NIH3T3 fibroblasts. NIH3T3 fibroblast is a widely studied model to represent peripheral clock, with regard to conserved rhythmic fluctuation of clock transcripts when compared with several other tissues [24,25]. Although the conservation of oscillatory nature of clock transcripts does not guarantee for equivalent functional properties, NIH3T3 can be used as a tool to pinpoint circadian transcript. The expression profile of *Per3* was used as the positive control.

To compare the expression of *EMT* in airway smooth muscle cells and vascular smooth muscle cells, the expression profile of *EMT*, *ACTA2* and *GAPDH* were extracted from two published microarray datasets (GEO accession GSE2883, GSE13168), which represent the transcription pattern of human vascular smooth muscle (VSM) cells and human airway smooth muscle (ASM) cells, respectively. These two datasets were established with the use of a same array platform (Affymetrix Human Genome U133A Array). The relative abundance of *EMT* was expressed after normalization to the expression of *ACTA2* or *GAPDH* in each group. Results were from three to four experimental replicates.

### Statistical analysis

All data were expressed in mean ± standard error (SEM). Statistics was analyzed by Student’s *t*-test and multiple comparisons were made by one-way analysis of variance (ANOVA). A *p* value less than 0.05 was considered statistically significant.

## Results and Discussion

### Cortisol exerts its permissive action at the plasma membrane level

**Figure 1:**
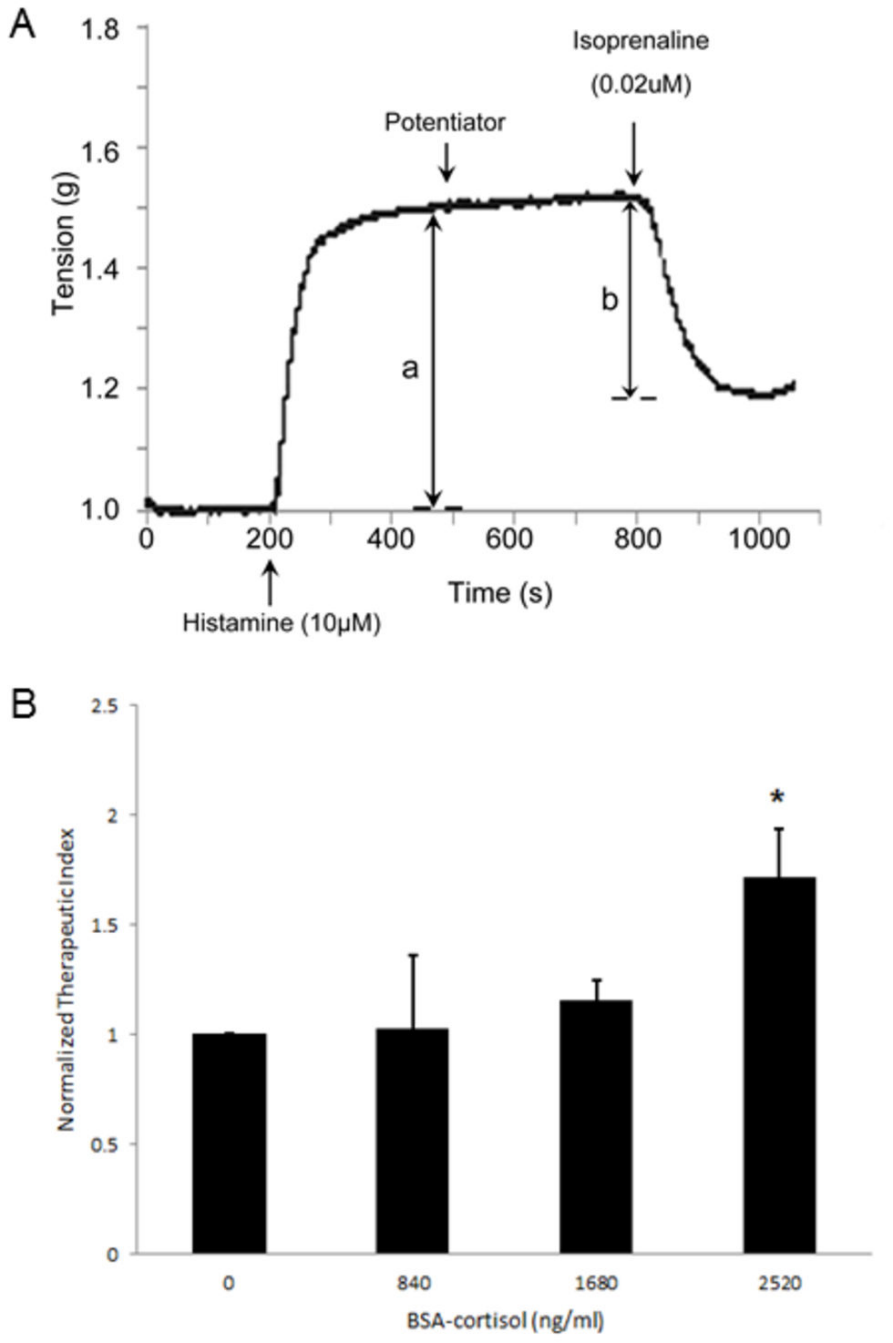
Cortisol exerts its permissive action at the plasma membrane level. ***A***, a schematic record of the tension curve. Histamine and isoprenaline were used to contract and relax the tracheal spiral, respectively. Each of the potentiators (including cortisol) was added to this system when contraction reached the plateau level. *B*, the relaxation effect of isoprenaline at different concentration of BSA-cortisol. The concentration gradient ranged from 0, 840, 1680 to 2520 ng/ml. The way to calculate therapeutic index and normalization were described in *Materials* and *methods*. * *p*<0.05 when 2520 ng/ml cortisol group is compared with baseline group (n=4 in each group).

To delineate the molecular mechanism of permissive action with clinical relevance, we first adopted a well-established *ex vivo* model [12]. Histamine, an important mediator of bronchoconstriction in asthma, was used to constrict the guinea pig tracheal spiral. Relaxation was induced by isoprenaline, an agonist for β-adrenoceptor (β-AR). Cortisol, when added after bronchoconstriction, potentiated the isoprenaline-induced bronchodilatation, a phenomenon consistent with the classical definition of permissive action. Therapeutic indices were calculated to quantify the relaxation effect (Figure 1A).

There is growing body of evidence to suggest that the rapid action of glucocorticoids is mediated by specific “receptors” localized to the plasma membrane. To provide further evidence, experiments were performed with membrane-impermeable steroids, namely BSA-conjugated cortisol (BSA-cortisol). Recently, we noticed a concentration threshold, only above which cortisol could amplify the action of isoprenaline [12]. To see whether it was also applicable for BSA-cortisol, we set a concentration gradient (840, 1680 and 2520 ng/ml), a series of dosages equivalent to cortisol of 100, 200 and 300 ng/ml, respectively, as the chemical linker and linkage stoichiometry (1:25) were well-defined for this commercially available preparation.

As expected, BSA-cortisol recapitulated the permissive action, but a higher concentration was needed if paralleled with cortisol (Figure 1B). As we have already probed the possible role of classical glucocorticoid receptor (GR) with the use of RU486 [12], a probable explanation for this observation could be that membrane “receptor” of cortisol was not the sole target for the permissive action. Even so, not all the cortisol molecules conjugated to BSA could exist in a suitable steric configuration to interact with its membrane target and then initiate downstream signaling.

### EMT shows robust expression in chondrocytes of guinea pig tracheal cartilage

**Figure 2:**
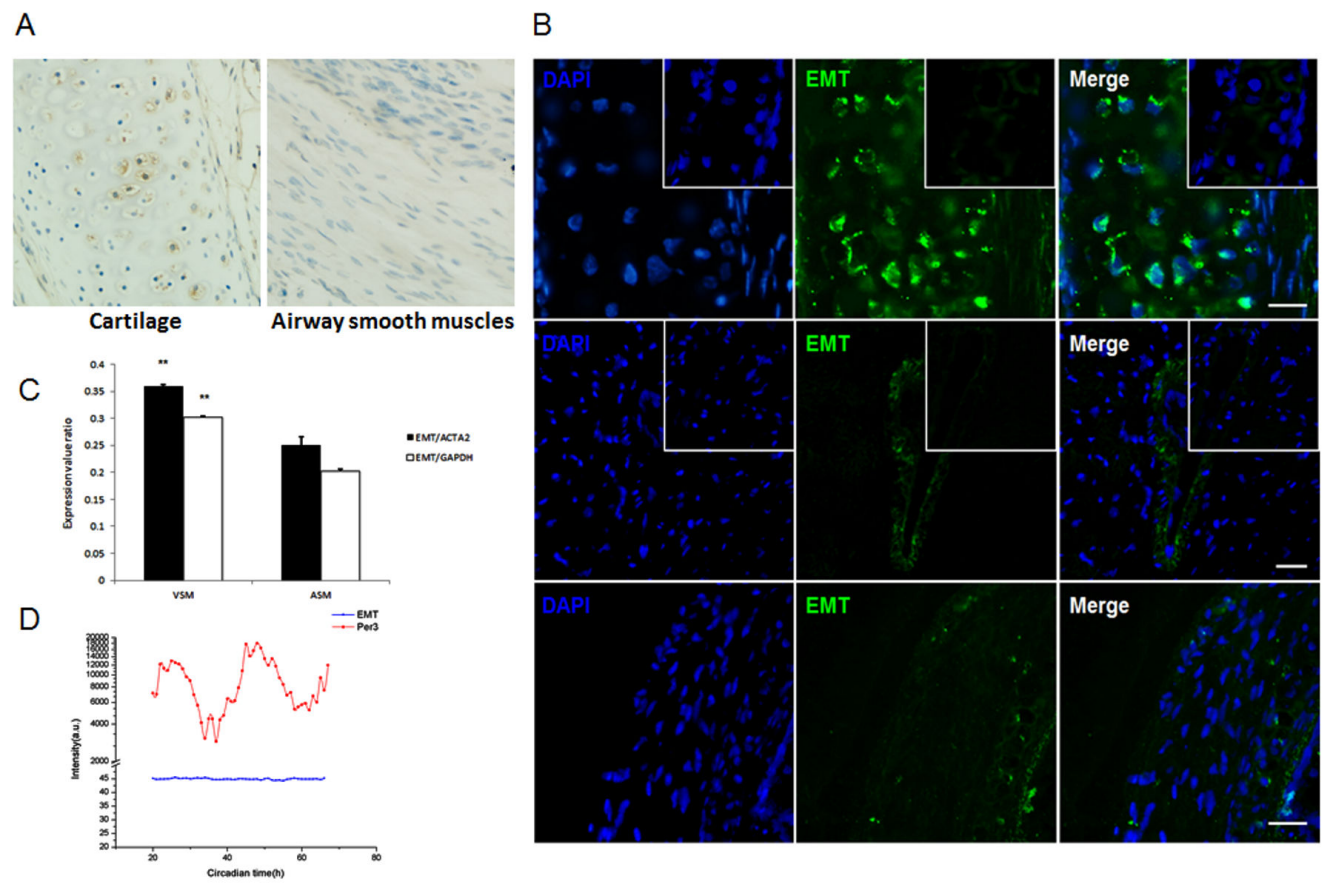
The expression prolife of EMT. *A*, immunohistochemical staining showed the expression of EMT in chondrocytes of tracheal cartilage, but not in airway smooth muscles. *B*, immunofluorescent labeling of EMT on chondrocytes (upper three subpanels), coronary smooth muscle cells (middle three subpanels) and airway smooth muscle cells (lower three subpanels). Specificity of EMT-immunolabeling was documented by antibody preabsorption (insets in upper and middle subpanels). The nuclei were stained blue with DAPI, and EMT was stained green by the immunofluoresence method. Scale bar: 20 μm. *C*, comparison of *EMT* expression in human airway smooth muscle (ASM) cells and vascular smooth muscle (VSM) cells. Data were extracted from open-access microarray datasets (GEO accession GSE2883, GSE13168). The expression values were normalized to *GAPDH* or *ACTA2* in each dataset, ** *p*<0.001. *D*, the circadian transcription patterns of EMT and Per3. Note that Per3 (a typical circadian gene) showed circadian fluctuation in transcription, while EMT transcription did not show circadian fluctuation. Data were extracted from an open-access microarray dataset (GEO accession GSE 11922).

EMT expression in guinea pig trachea has not previously been established. Hence the spatial expression pattern of EMT was evaluated by immunohistochemical and immunofluorescent staining in trachea rings. Surprisingly, we observed that EMT was highly enriched in the chondrocytes of tracheal cartilage (Figure 2A and 2B), but was undetectable in tracheal smooth muscle cells (Figure 2A and 2B). The specificity of EMT expression was further confirmed by its absence in the preabsorption control (Figure 2B, inset). As a positive control, EMT was expressed in the coronary smooth muscles of guinea pig (Figure 2B), further supporting the efficacy and specificity of EMT antibody. We cannot definitely exclude the expression of EMT in airway smooth muscles, which has been shown in the bronchial smooth muscle of FVB mice with the use of a self-prepared primary antibody [26], but suggest a more abundant expression in tracheal chondrocytes, at least detected by the commercially validated primary antibody used in this study. This unique expression pattern implies that chondrocytes of tracheal cartilage may play an important role in the permissive action of cortisol, while tracheal smooth muscles may not be the solely target of cortisol in its permissive effect.

We and others have suggested that the compromised bronchodilatation effect of β-agonist is due to low level of cortisol at 3–4 a.m. This oscillation of circulating cortisol correlates with nocturnal symptom of asthma, which can be recapitulated by the trachea model mentioned above [12]. Central and peripheral clock systems communicate with each other at multiple levels. Peripheral tissues may antagonize the central circadian clock by employing self-oscillating pacemaker, as glucocorticoid receptor is regulated by Clock-Bmal1 heterodimer [27]. Given that EMT acts as the presumable target of cortisol in our model system, we analyzed its temporal expression pattern from a microarray dataset (GEO accession GSE 11922). EMT transcripts did not show circadian fluctuation in this 1-hour resolution profiling, but displayed a steady expression at such high density sampling when compared with typical circadian molecule Per3 (Figure 2D). In aggregate, these observations indicate that EMT is a plausible target of cortisol, prompting us to further analyze its role in the trachea model of permissive action.

### Pharmacological inhibition of EMT imitates the permissive action

**Figure 3:**
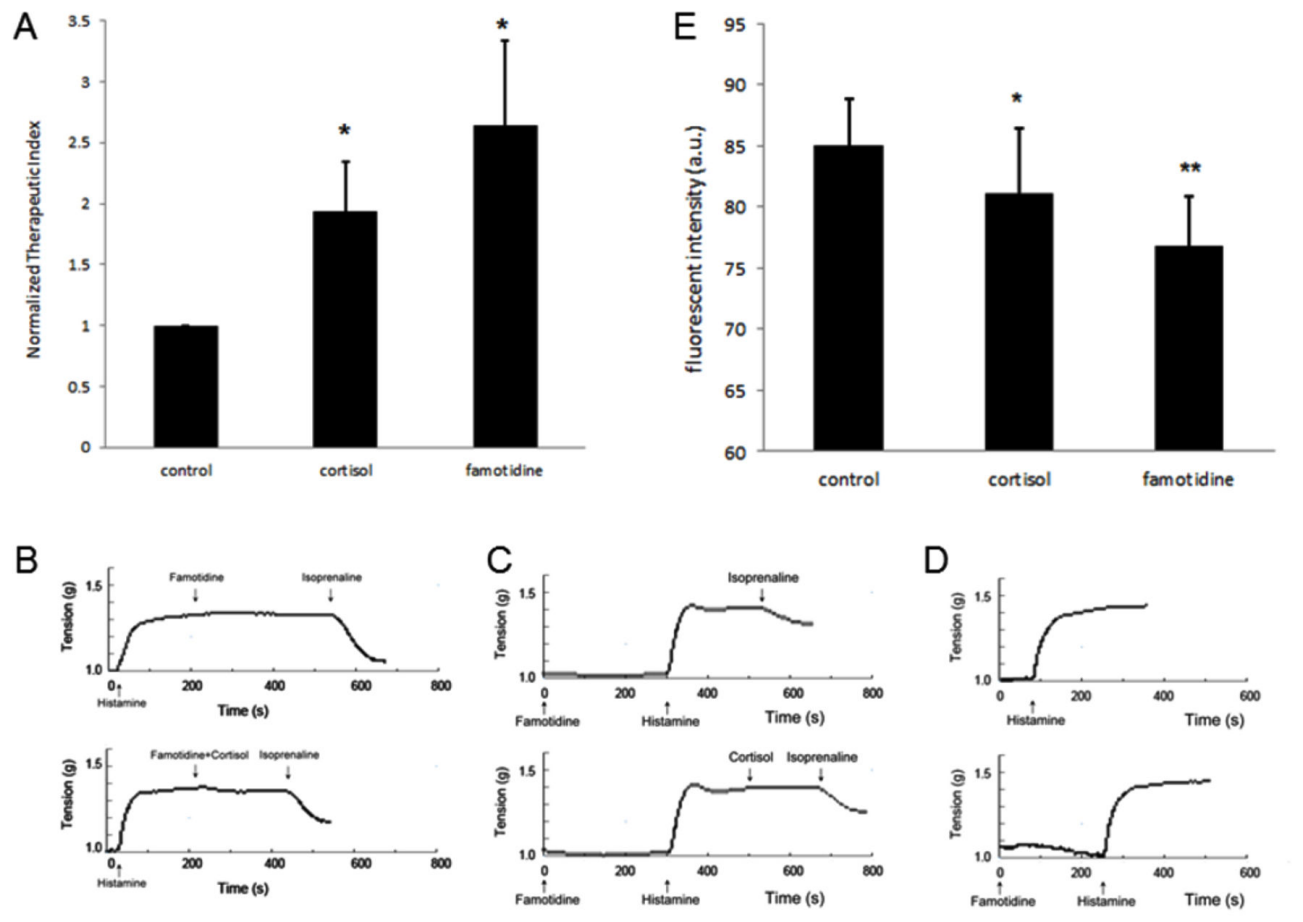
EMT inhibition potentiates the spasmolytic action of isoprenaline. *A*, famotidine, a potent EMT inhibitor, when added in the replacement of cortisol, achieved a more profound permissive effect for isoprenaline (n=3 in each group), * *p*<0.05 *vs*. control. *B*, there was no additive effect when famotidine and cortisol were administrated in sequential order. *C*, with famotidine preincubation, cortisol did not further potentiate the spasmolytic action of isoprenaline. ***D***, a similar contractile response was produced by histamine with or without famotidine pretreatment. *E*, SPG method to quantify catecholamine uptake by tracheal chondrocyte. Pretreatment with EMT inhibitors (cortisol or famotidine) hampered catecholamine uptake (50 μM norepinephrine, 20 min incubation). Fluorescent intensity was measured in approximately 50 cells for each group, * *p*<0.05, ** *p*<0.001 *vs*. control. a.u., arbitrary unit.

Next, we intended to clarify the precise role of EMT in the permissive action via pharmacological manipulation. Famotidine appears to be the most potent inhibitor for EMT yet identified, as determined at single cell level [28]. Not surprisingly, famotidine potentiated the relaxation effect of isoprenaline and the therapeutic index rose significantly even compared with cortisol (Figure 3A). Similar results were obtained with the use of cimetidine, another EMT inhibitor [29] (data not shown). A second challenge of tracheal spiral with cortisol subsequent to the primary stimulation with famotidine did not give rise to an additive effect (Figure 3B). In addition, the potentiation effect of cortisol on tracheal spiral could no more be observed if the tracheal spiral was pretreated with famotidine (Figure 3C). Supposing that EMT is the same target of these two agents, the first applied agent has already occupied the allosteric site for EMT inhibition in saturation, the second applied agent therefore could not exert its effect. This may be a reasonable explanation for these observations.

Although with the knowledge that type 1 histamine receptor (H1R) on the airway smooth muscle is responsible for the constrictive action of histamine [30], we still assessed the response of tracheal spiral to histamine in the presence of famotidine, a selective type 2 histamine receptor (H2R) antagonist. As supposed, similar contractile responses were elicited by histamine either with or without famotidine. This observation excludes the potential influence of famotidine on airway smooth muscle constriction via the H2R (Figure 3D).

In clinical scenario, the association between H2R antagonists and asthma has been aware for a long while. Most of the studies ascribed the amelioration of airway hyper-responsiveness to the anti-reflux effect of H2R antagonists on gastroesophageal tract, although some studies mentioned that the blockade of weak H2R signaling in airway tissue may also work in part. However, we noticed that several reports emphasized the influence of H2R antagonists on the disposition of theophylline in both healthy volunteers [31] and patients with airway disorders [32]. Although these results were thought to be related to CYP450-mediated metabolism, it could also be explained as impact on the xenobiotic transporter, such as EMT. Whether theophylline is a substrate of EMT needs further investigations.

### EMT inhibition slows down catecholamine uptake by chondrocytes

**Figure 4:**
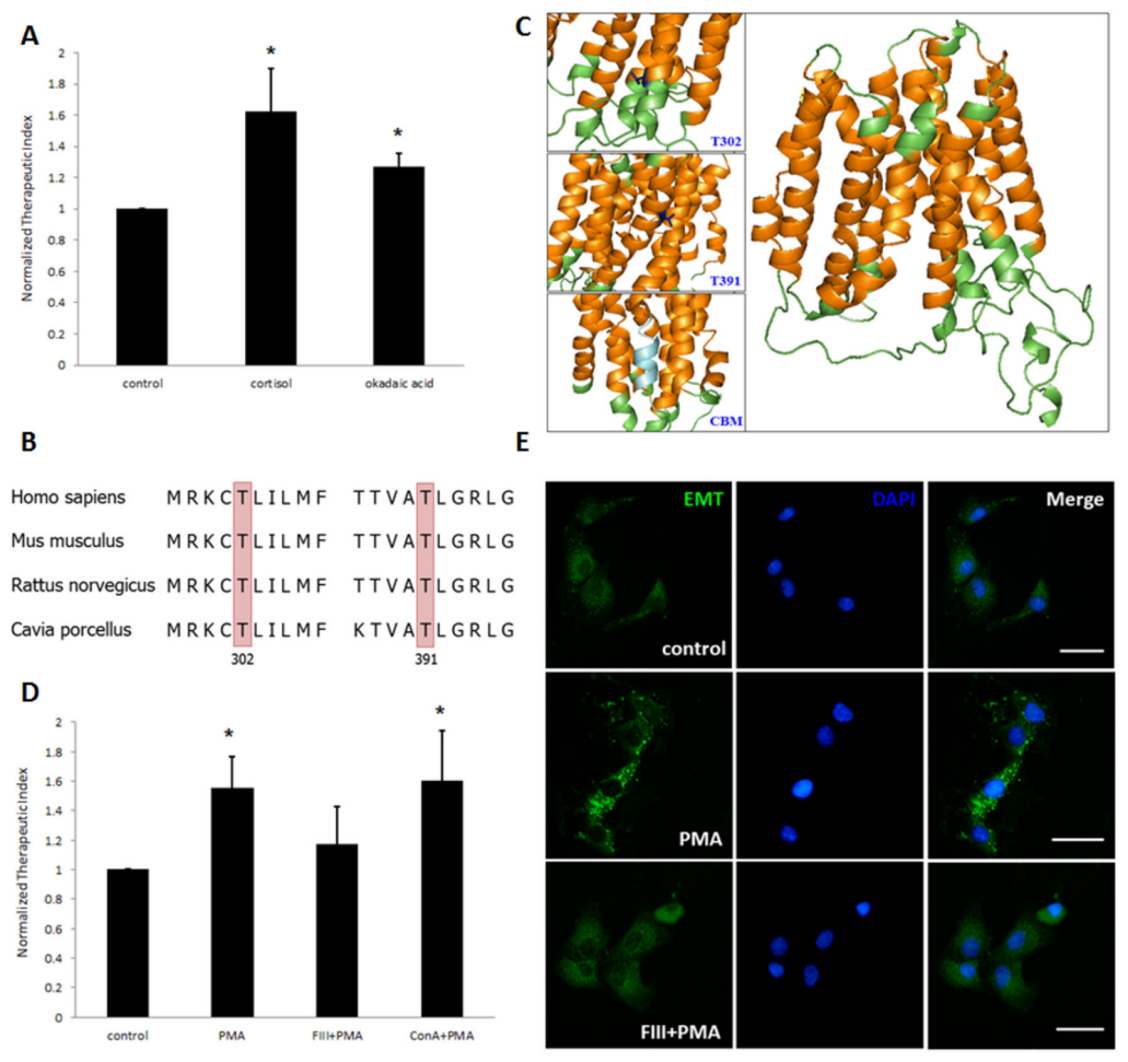
Protein kinase C (PKC) phosphorylates EMT and induces EMT internalization. *A*, okadaic acid, an inhibitor of protein phosphatase PP1/2A, partially imitated the permissive action of cortisol (n=3 in each group), * *p*<0.05 *vs*. control. *B*, bioinformatics analyses of guinea pig EMT amino acid sequence revealed threonine 302 and 391 as consensus PKC site, which were also conserved across species. *C*, an overall view of predicated guinea pig EMT, showing transmembrane (TM) helices in orange. Other residues were represented in green. Two consensus PKC site, one located at juxtamembrane region (Thr302) and the other in TM helix were depicted in blue (upper and middle insets). A typical caveolin-binding motif (CBM) was also shown in silver (lower inset). *D*, endocytic inhibitor filipin III (FIII), but not concanavalin A (ConA) blocked the permissive effect induced by PKC activator, PMA (n=4 in each group). *E*, immunofluorescent staining of EMT in vascular smooth muscle cells with different pharmacological manipulation (upper three subpanels, control; middle three subpanels, PMA treatment; lower three subpanels, filipin III exposure and then PMA treatment). The nuclei were stained blue with DAPI, and EMT was stained green by the immunofluoresence method. Scale bar: 20 μm.

To provide further evidence that EMT is the functional transporter in our model, we applied a more direct approach to assess the uptake process of catecholamine by chondrocytes in tracheal rings. Intracellular catecholamine can be measured using a sucrose-potassium phosphate-glyoxylic acid (SPG) method, in which highly active aldehyde (glyoxylic acid) is used to react with monoamines and produce fluorescent compounds under suitable illumination. This method was validated in vascular smooth muscle cells (Figure S1), which were shown to express EMT (Figure 4E). And pharmacological inhibition of EMT did abolish the uptake of catecholamines (Figure S1). After determined the reliability of SPG method at the cellular level, we then used the same method to detect the uptake of catecholamine in tracheal tissues. As expected, fluorescence was observed in chondrocytes after exposure the tracheal rings to catecholamine. Pretreatment with cortisol or famotidine, both EMT inhibitors, decreased the intracellular fluorescence (Figure 3E). No obvious fluorescence was observed in airway smooth muscles.

Taken together, our observations indicate that EMT-enriched chondrocytes are functional sites for catecholamine uptake, and pharmacological inhibition can imitate the permissive effect by slowing down the uptake process.

### EMT phosphorylation and internalization accounts for the role of PKC in the permissive action

Accumulating evidence has suggested that membrane-initiated steroid signaling (MISS) underlies the acute action of glucocorticoids [33]. After demonstrating the involvement of membranous EMT in our model, we further probed the role of MISS, which also functions at the level of plasma membrane. Downstream from the putative membrane receptors, diverse signaling pathways have been shown to mediate the rapid action of glucocorticoids in different cell types, G_q_-phospholipase C (PLC)-protein kinase C (PKC) is one of them [34]. We have already demonstrated the critical role of PKC activation in the permissive action of cortisol, showing that pharmacological blockade of PKC partially abolished the permissive action of steroid [12]. To coordinate the roles of PKC and EMT, we postulated that PKC, a serine/threonine kinase, phosphorylates EMT. Inhibition of corresponding phosphatases could achieve similar results as activation of specific kinase. Similar to the effect of phorbol-12-myristate-13-acetate (PMA) treatment, okadaic acid, an inhibitor of protein phosphatases PP1/2A, also potentiated the relaxation effect of isoprenaline (Figure 4A). This post-translational modification (PTM) might exert a regulatory effect on membranous EMT. Analyses of guinea pig EMT amino acid sequence by NetPhos (www.cbs.dtu.dk/services/NetPhosK) and Scansite (scansite.mit.edu) programs revealed Thr302 and Thr391 as potential consensus PKC phosphorylation sites. Further phylogenic comparison indicated that these two sites were evolutionary conserved across species, suggesting their importance for biological fitness (Figure 4B). We created a three-dimensional structure of guinea pig EMT based on RaptorX web server (raptorx.uchicago.edu), a newly developed web-based method delivering high-quality structural models for targets even with remote template [35]. In our model, these two predicted phosphorylation sites were located at different positions. Thr302 was situated in the juxtamembrane region at the cytosolic side of EMT, while Thr321 was embedded in the plasma membrane (Figure 4C). Therefore, it appears that only threonine at position 302 is in an exposed state for PKC accession and phosphorylation. Our data implicate that EMT phosphorylation is a possible mechanism to explain the role of PKC in the permissive effect. As there is no commercially available antibody to recognize phosphor-specific-EMT, more proper biochemical studies are needed to provide direct proofs of EMT phosphorylation, which are beyond the scope of this article.

PKC has been shown to regulate the reuptake transporters of various neurotransmitters. Relative to EMT, the main transporter for catecholamine clearance in central nerve system, NET, is also regulated by PKC via cytosolic residues phosphorylation [36]. This PTM triggers a rapid sequestration of the transporters from plasma membrane via clathrin-independent endocytosis [37]. Accordingly, phosphorylation induced internalization may also be true for EMT. As a consequence, loss of membrane localization leads to decreased amount of catecholamine reuptake, which could explain how PKC activation results in adrenergic signaling potentiation. To test this hypothesis, different inhibitors of endocytic pathway were used in the present study. Disruption of clathrin-mediated endocytosis by concanavalin A (ConA) did not prevent the permissive effect of PMA (a PKC activator). As cholesterol-disrupting agents do not interact with cholesterol in clathrin-coated pits, they are ideal tools to distinguish the involvement of clathrin pathway from that of lipid raft/caveolae pathways [38]. Treatment of the tracheal spiral with filipin III, a lipid-raft-disrupting agent, abolished the PKC-mediated permissive action (Figure 4D). Collectively, these results demonstrated that lipid raft/caveolae, but not clathrin, was involved in PKC-induced permissive effect. Moreover, the punctate pattern of EMT expression in chondrocyte also implicates the localization of this 12-transmembrane protein on the endocytic vesicles (Figure 2B), which was further confirmed in vascular smooth muscle cells (Figure 4E, upper three subpanels). The punctate pattern of EMT expression was enhanced after PMA treatment (Figure 4E, middle three subpanels) and this change could be abolished with the pre-exposure to filipin III (Figure 4E, lower three subpanels). Retrospectively, we mapped a typical caveolin-binding motif (CBM) (φXφXXXXφ, where φ is an aromatic amino acid and X is any amino acid) in its mirror form at the fifth transmembrane helix in the predicted EMT structure [39], which is consistent with the membrane-inserted topology of caveolin (Figure 4C).

### EMT-centered pathway is associated with airway disorders in human

**Figure 5:**
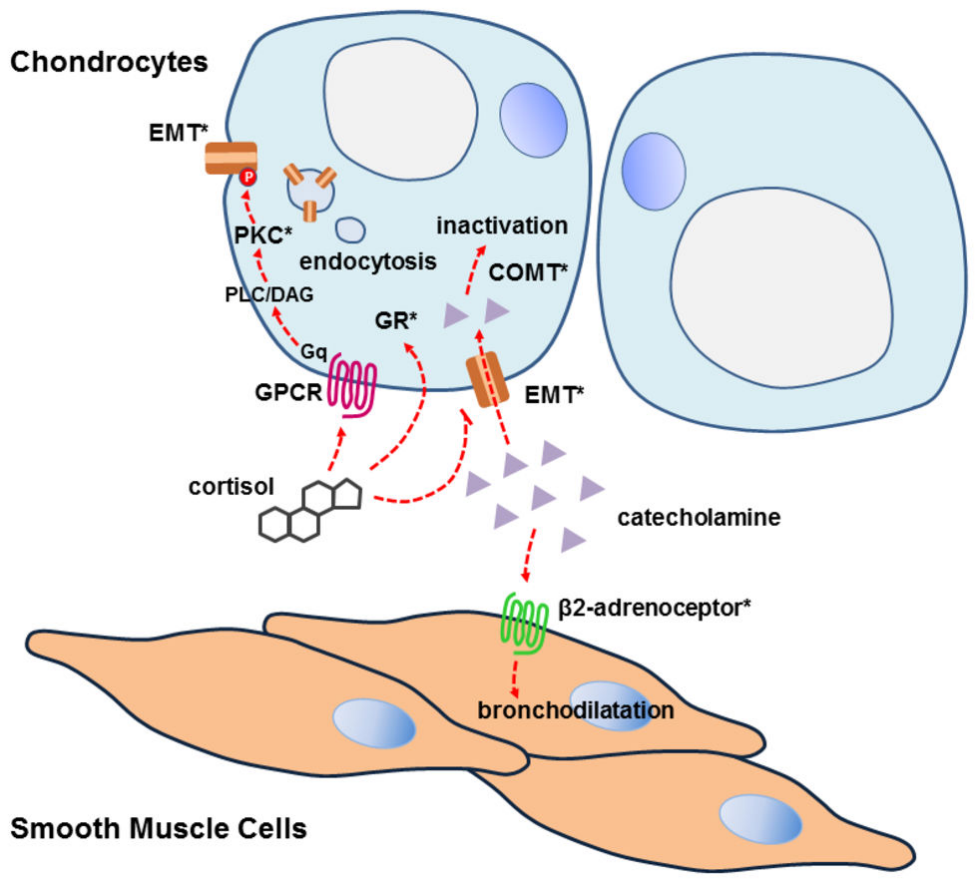
A putative EMT-centered model explains the permissive action of cortisol in trachea. EMT as the direct target is inhibited by cortisol, and this action slows down the reuptake process of catecholamines by chondrocytes, leading to extracellular catecholamines accumulation and more considerable adrenergic signaling activation in smooth muscle cells. Moreover, cortisol-initiated membrane signaling triggers PKC activation, which phosphorylates EMT and results in EMT internalization. Signaling components associated with airway disorders in genetic studies are labeled with asterisks (*).

Finally, we tried to make a clinical association of glucocorticoid nongenomic pathway with human diseases, especially airway disorders. Based on our data, a novel model was proposed to explain the non-genomic action of cortisol in trachea and the interaction between chondrocytes and smooth muscle cells was highlighted (as summarized in Figure 5). An approach to identify the biological signaling in a disease (such as asthma) is to look not at a single gene, but rather sets of genes. In this aspect, we scrutinized major components in our model for their involvement in clinical conditions. EMT, as the central part of this model, has been demonstrated to correlate with the severity of asthma in a recent report [40]. For β2-adrenergic receptor, the effecter of bronchodilatation, at least two polymorphisms (Arg16Gly and Gln27Glu) have been studied extensively for their association with asthma and related phenotypes [41]. Even COMT, the cardinal enzyme for inactivation of endogenous and exogenous catecholamine, was mapped as a frequently altered locus in obstructive airway disease [42]. To our surprise, aldehyde dehydrogenase (ALDH), the essential enzyme for metabolic disposition of both ethanol and catecholamine, showed genotype bias in asthmatic subjects [43]. Nevertheless, ALDH dysfunction-related asthma was thought attributed to inadequate metabolism of aldehyde, the latter elicited bronchial asthma as an allergen [44]. With caution, we did not include ALDH in our model. Hitherto, in combination with the two components (GR and PKC) formerly assessed [12], at least five members in our model have been reported as susceptible loci underlying airway disorders. To the best of our knowledge, this was the first time to incorporate multiple variants confirmed in different studies into a same signaling pathway, which may be unrecognized for airway disease pathogenesis.

As integral parts of many physiological processes, catecholamines and their signaling have attracted extensive research in the past century. These studies have provided detailed information on how catecholamines produce, release and initiate downstream signaling. However, less attention has been paid for how these processes are ceased, especially for the role and regulation of several reuptake transporters. Our findings unequivocally demonstrate that EMT is the direct target of cortisol in its permissive action. The inhibition of EMT by cortisol slows down the reuptake process, leading to extracellular catecholamines accumulation and more considerable adrenergic signaling activation in specific effecter cells. Moreover, cortisol-initiated membrane signaling triggers PKC activation, which phosphorylates EMT and results in redistribution of this transporter from the plasma membrane.

A critical finding in our study is a much stronger expression of EMT in tracheal chondrocytes than smooth muscle cells. The expression of EMT in smooth muscle cells has been documented in several reports, mainly in vascular smooth muscle (VSM) cells [20,45,46]. We cannot fully deny the expression of EMT in airway smooth muscle (ASM) cells. But in consideration of the different transcriptome between ASM and VSM cells, there can be a more abundant expression of EMT in the latter cell type. Indeed, with the use of publicly available microarray datasets, we observed 1.5-fold upregulation of *EMT* in VSM than ASM cells, after normalization of the expression value to either *GAPDH* or *ACTA2* (Figure 2C). And the detection of EMT expression in vascular smooth muscles (coronary artery in this study) was also justified via immunofluorescence assay (Figure 2B). We further confirmed the functionality of chondrocyte-expressed EMT by measuring the uptake of catecholamine (Figure 3E). While Horvath and colleagues noticed the uptake of long-acting β2-agonists by primary human bronchial smooth muscle cells, which was thought to be mediated by EMT [47]. In their experiment, radiolabeled long-acting β2-agonists at a concentration of nanomolar were used as the substrates. But for aldehyde-based histochemical method, it can only differentiate catecholamine at ten to hundreds micromolar [17,20]. Thus, we just conclude that EMT is highly enriched in guinea pig tracheal chondrocytes, and is functional. Further investigations are needed to fully differentiate the contribution of ASM- and chondrocyte-expressed EMT for catecholamine clearance, and the role of chondrocyte-expressed EMT should also be evaluated in other species.

Taken together, our data stress the novel and pivotal role of cartilaginous EMT in the permissive action of cortisol. In a practical aspect, regulations of EMT activities can be achieved by at least two distinct strategies: (i) allosteric inhibition of EMT and (ii) PTM triggered internalization of EMT. This EMT-centered signaling may be critical for airway physiology and pathophysiology.

## Conclusions

The present study supports that cortisol realizes its permissive action via both direct inhibition and PKC-dependent EMT phosphorylation and thereafter internalization. This inhibitory effect of cortisol on EMT results in accumulation of extracellular catecholamines and stronger adrenergic signaling, and thus potentiates the bronchodilatating action of catecholamines. The finding that EMT mediates the intimate crosstalk between steroids and adrenergic signaling may shed light on the development of new interventions for airway disorders. The study also demonstrates for the first time that cartilaginous EMT, rather than smooth muscle EMT, plays a critical role in tracheal catecholamine metabolism and function. This finding may open up a new direction in cartilage research.

## Supporting Information

Figure S1 **SPG method to quantify catecholamine uptake by rat vascular smooth muscle cells.** Pretreatment with EMT inhibitors (cortisol or famotidine, 5 min) hampered catecholamine uptake (50 μM norepinephrine, 20 min incubation). Fluorescent intensity was measured in approximately 50 cells for each group, ** *p*<0.001 *vs*. control. a.u., arbitrary unit. Scale bar, 20 μm.
(TIF)
