# Supplementary figures and images for: Extraneuronal Monoamine Transporter Mediates the Permissive Action of Cortisol in the Guinea Pig Trachea: Possible Involvement of Tracheal Chondrocytes
Source: PLoS One. 2013 Oct 1;8(10):e76193. doi: 10.1371/journal.pone.0076193 (PMC3787990; doi:10.1371/journal.pone.0076193)

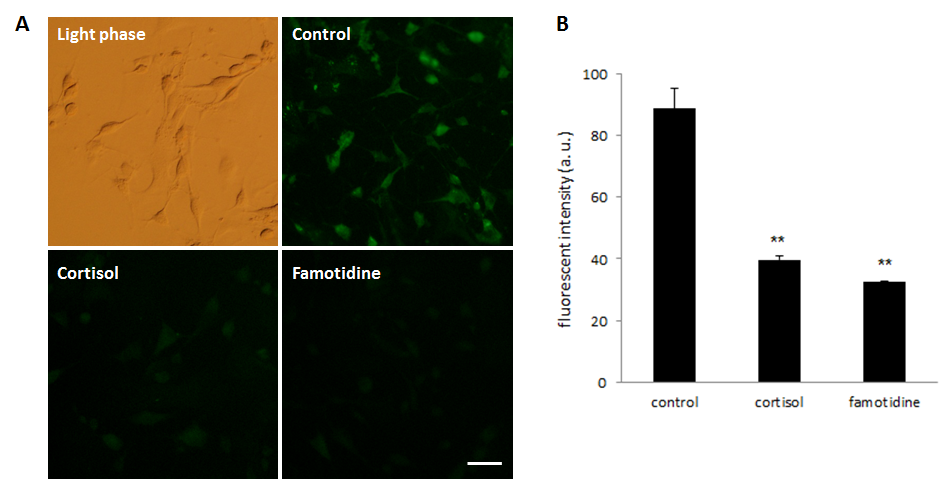

Supplement: Figure S1 — SPG method to quantify catecholamine uptake by rat vascular smooth muscle cells. Pretreatment with EMT inhibitors (cortisol or famotidine, 5 min) hampered catecholamine uptake (50 μM norepinephrine, 20 min incubation). Fluorescent intensity was measured in approximately 50 cells for each group, ** p<0.001 vs. control. a.u., arbitrary unit. Scale bar, 20 μm. (TIF) [file pone.0076193.s001.tif]
